# Expanding the list of sequence-agnostic enzymes for chromatin conformation capture assays with S1 nuclease

**DOI:** 10.1186/s13072-023-00524-4

**Published:** 2023-12-11

**Authors:** Gridina Maria, Popov Andrey, Shadskiy Artem, Torgunakov Nikita, Kechin Andrey, Khrapov Evgeny, Ryzhkova Oxana, Filipenko Maxim, Fishman Veniamin

**Affiliations:** 1 Institute of Cytology and Genetics SB RAS, Novosibirsk, Russia; 2 Novosibirsk State University, Novosibirsk, Russia; 3 Institute of Chemical Biology and Fundamental Medicine, Novosibirsk, Russia; 4 Research Centre for Medical Genetics, Moscow, Russia; 5 Artificial Intelligence Research Institute, AIRI, Moscow, Russia

## Abstract

This study presents a novel approach for mapping global chromatin interactions using S1 nuclease, a sequence-agnostic enzyme. We develop and outline a protocol that leverages S1 nuclease's ability to effectively introduce breaks into both open and closed chromatin regions, allowing for comprehensive profiling of chromatin properties. Our S1 Hi–C method enables the preparation of high-quality Hi–C libraries, marking a significant advancement over previously established DNase I Hi–C protocols. Moreover, S1 nuclease's capability to fragment chromatin to mono-nucleosomes suggests the potential for mapping the three-dimensional organization of the genome at high resolution. This methodology holds promise for an improved understanding of chromatin state-dependent activities and may facilitate the development of new genomic methods.

**Supplementary Information:**

The online version contains supplementary material available at 10.1186/s13072-023-00524-4.

## Introduction

Genomic assays, which profile chromatin properties on a whole-genome scale, rely significantly on enzymatic activities corresponding to different chromatin states. For instance, MNase-seq assay utilizes micrococcal nuclease's preferential digestion of nucleosome-free regions, while DNase hypersensitivity assay exploits DNase I enzyme's preferential cutting in open chromatin regions [1]. The discovery of transposase Tn5 and similar enzymes, which also exhibit affinity to open chromatin regions, has further simplified the analysis of open chromatin [2]. Therefore, expanding the repertoire of enzymes with specific, chromatin-state-dependent activities is crucial for developing novel genomic methods.

In certain applications, enzymes with activity limited to specific chromatin states are necessary, while in others, uniform genomic coverage is essential. Chromatin conformation capture assays, including its whole-genome modification known as Hi–C, notably benefit from using enzymes with sequence-agnostic chromatin cleavage. Hi–C has been utilized by others and us to study genome architecture across various tissues [3] and individual cells [4], understand mechanisms underlying gene regulation in development [5, 6], explore alterations in gene expression in cancer [7], assemble genomes [8, 9], and identify structural variants [10].

The Hi–C technique involves several mandatory steps: cell fixation with formaldehyde to preserve native genome folding in the nucleus, chromatin fragmentation, chromatin end labeling by biotin, and chromatin ligation [11]. Consequently, fragments in close spatial proximity are ligated, and a biotin label is employed to selectively enrich these ligated fragments.

Enzymes for chromatin fragmentation in an Hi–C experiment can vary depending on the study's purpose. Initial protocols recommended using restriction enzymes with a 6 bp recognition site, limiting data resolution to approximately 10 kb [12]. However, the introduction of 4-base cutters and other modifications significantly improved this resolution [13]. Genome-wide distribution of restriction sites and sequencing depth are the limiting factors for resolving fine-scale chromatin organization. These limitations can be circumvented using a mixture of restriction enzymes or sequence-agnostic nucleases for chromatin fragmentation [14–16]. Currently, two sequence-agnostic enzymes, MNase and DNase I, are used in Hi–C studies. MNase Hi–C can generate nucleosome-level interaction maps and is proficient in loop detection, although it is less apt for compartment detection than conventional Hi–C [17]. Using DNase I Hi–C protocol improves data resolution and genomic coverage over conventional Hi–C [15]. However, DNase I Hi–C libraries contain a high level of non-informative fragments or "dangling ends" (DE) [11], necessitating deeper sequencing.

S1 nuclease, a sequence-agnostic enzyme, degrades single-stranded nucleic acids and cleaves nick, gap, mismatch or loop structures in double-stranded DNA. S1 nuclease can also introduce breaks into double-stranded DNA at high enzyme concentrations [18]. S1 nuclease's ability to cleave unpaired DNA is exploited in S1-seq and S1-END-seq for studying DNA secondary structure and meiotic double-strand break end resection on a genome-wide scale [19–21].

In this study, we delineate a profile of chromatin digestion by S1 nuclease and establish a protocol utilizing this enzyme for mapping global chromatin interactions. Our findings reveal that S1 nuclease effectively introduces breaks into both open and closed chromatin. The developed S1 Hi–C method enables the preparation of high-quality Hi–C libraries, surpassing the performance of the previously published DNase I Hi–C protocol. Furthermore, S1 nuclease fragments chromatin to mono-nucleosomes, implying that the three-dimensional organization of the genome can be mapped with high resolution.

## Methods

### Experimental procedures

#### Human cells isolation and culture

Human K562 cells were grown in RPMI-1640 medium with 10% FBS and a penicillin/streptomycin mix. The cells were collected, washed in PBS to remove traces of serum, resuspended in PBS at a concentration of 1 × 10^6^ cells/ml and processed immediately for crosslinking.

Human peripheral blood mononuclear cells (PBMCs) were isolated from peripheral blood samples, which were collected in tubes with EDTA. RBC lysis buffer (BioLegend) was used for erythrocyte removal according to the manufacturer’s instructions. Isolated PBMCs were washed in PBS, cells were counted and resuspended in PBS at a concentration of 1 × 10^6^ cells/ml and processed immediately for crosslinking.

Human skin fibroblasts were maintained in DMEM/F12 with 10% FBS and a penicillin/streptomycin mix (all from Invitrogen). The cells were disaggregated by a trypsin (Invitrogen) treatment, washed in PBS, resuspended in PBS at a concentration of 1 × 10^6^ cells/ml and processed immediately for crosslinking.

Human induced pluripotent stem cells (iPSCs) were maintained under feeder-free culture conditions on Matrigel Matrix coated dishes in mTeSR™1 medium. The cells were disaggregated by a TrypLE (Invitrogen) treatment, washed in PBS, resuspended in PBS at a concentration of 1 × 10^6^ cells/ml and processed immediately for crosslinking.

#### S1 Hi–C protocol

1. Cells were fixed by adding formaldehyde solution (F8775, Sigma-Aldrich) to a final concentration of 1% and incubating for 10 min at room temperature (RT) with continuous rotation. Crosslinking was quenched by adding 2.5 M glycine to a final concentration of 0.125 mM and incubating for 10 min at RT with continuous rotation. The cell suspension was centrifuged at 1100 g for 10 min, resuspended in PBS and split into aliquots of 2.5 × 10^6^ cells. The cells were centrifuged at 1100 g for another 10 min. The cell pellets were snap-frozen and stored at − 80 °C. [11]
2. Cell lysis and chromatin fragmentation
  2.1 An aliquot of cross-linked cells was placed on ice and gently resuspended in 1 ml of cell lysis buffer (10 mM Tris–HCl pH 8.0, 10 mM NaCl, 0.5% Igepal).
  2.2 The cells were incubated for 1 h with intermittent rotation at RT.
  2.3 Centrifugation was performed at 2500 g for 5 min.
  2.4 The supernatant was removed, and the pellet was gently resuspended in 200 μl of DNase I buffer (50 mM Tris–HCl pH 7.5, 0.5 mM CaCl_2_) and 0.2% SDS.
  2.5 The cells were incubated at 37 °C for 1 h.
  2.6 Control point #1: 5 μl of lysed cells were saved to check gDNA integrity.
  2.7 SDS was quenched by adding 25 μl of 10% Triton X-100 10 min at 37 °C.
  2.8 Centrifugation was performed at 2500 g for 5 min.
  2.9 Pellet was resuspended in 500 μl of 1 × S1 nuclease buffer (Thermo Scientific) with 1% Triton X-100.
  2.10 Centrifugation was performed at 2500 g for 5 min.
  2.11 Pellet was resuspended in 80 μl of 1 × S1 buffer
  2.12 S1 nuclease (200 U; Thermo Scientific) was added and incubated at 37 °C for 1 h.
  2.13 The reaction was stopped by adding 5 μl of 500 mM EDTA.
  2.14 Control point #2: 5 μl of the reaction was saved to check the S1 nuclease digestion efficiency. 90 µl of lysis buffer (10 mM Tris–HCl pH 8.0, 10 mM NaCl, 0.3% SDS) and 5 μl of proteinase K (800 units/ml) were added to both controls. The controls were reverse cross-linked at 65 °C for at least 8 h. DNA was extracted by the standard phenol–chloroform method.
  2.15 Reaction was purified by 0.8 volume of AMPure beads according to the manufacturer's recommendations.
  2.16 The beads were resuspended in 100 μl of 1 × NEBuffer 3.1, and the AMPure Beads remained in the mixture.
  2.17 The sample was put on ice.
3. Biotin labelling
  3.1 100 μl of fill-in master mix (1 × NEBuffer 3.1; 150 μM each: dATP, dTTP, dGTP, Biotin-15-dCTP; 50U DNA Polymerase I, Large (Klenow) Fragment) was added to the sample from the step 2.17.
  3.2 Reaction was incubated at 23 °C for 4 h with intermittent gentle shaking.
4. In situ ligation
  4.1 800 μl of ligation mix (1 × T4 ligase buffer (Neb); 1.25% Triton X-100, 6.25% PEG 8000, 12.5 mM BSA, 20 μl T4 DNA ligase) was added to the sample from the step 3.2.
  4.2 The reaction was incubated at 16 °C for at least 8 h (overnight is also appropriate) with continuous shaking.
5. Cross-link reversal
  5.1 The reaction was centrifuged at 2500 g for 5 min.
  5.2 The supernatant was discarded, and the pellet was resuspended in 400 μl of lysis buffer (10 mM Tris pH 8.0, 10 mM NaCl, 0.3% SDS).
  5.3 20 μl of proteinase K (800 units/ml) was added to the mixture.
  5.4 The reaction was incubated at 65 °C for 4 h with vigorously shaking.
  5.5 Another 20 μl of proteinase K (800 units/ml) was added.
  5.6 The mixture was incubated at 65 °C for another 4 h (overnight is also appropriate) with vigorously shaking.
  5.7 3 μl of Glycoblue, 50.5 μl of 3 M NaAc and 506 μl of isopropanol were then added.
  5.8 The mixture was incubated at − 80 °C for 20 min.
  5.9 The mixture was centrifuged at 16,000 g for 40 min at 4 °C.
  5.10 The supernatant was discarded, and the DNA and AMPure Beads pellet was resuspended with 100 μl of nuclease-free water containing 5 μg of RNase A.
  5.11 The resuspended pellet was incubated at 37 °C for 30 min with continuous shaking.
  5.12 DNA was purified by 1 volume of AMPure beads according to the manufacturer's recommendations.
  5.13 The concentration of the recovered DNA was measured with a Qubit fluorometer. The yield was 3–6 μg if starting with 2.5 × 10^6^ cells.
6. Removal of biotin from unligated ends
  6.1 Purified DNA was treated with 5 U T4 DNA polymerase (Neb) in a 100-μl reaction containing 1 × NEBuffer 2.1, 12 μM of each dATP and dGTP at 20 °C for 90 min.
  6.2 The reaction was stopped by adding 5 μl of 500 mM EDTA.
  6.3 DNA was purified by 1 volume of AMPure beads according to the manufacturer's recommendations.
  6.4 The concentration of the recovered DNA was measured with a Qubit fluorometer.
7. DNA fragmentation, end-repairing, A-tailing and adapter ligation were prepared using KAPA HyperPlus Kits (Roche) according to the manufacturer's recommendations. 1 μg of DNA from the step 6.3 was used as input.
8. Biotin pulldown
  8.1 Dynabeads® MyOne™ Streptavidin C1 (30 μl per reaction) were washed twice with 1 × B&W buffer (5 mM Tris–HCl pH 8.0, 0.5 mM EDTA, 1 M NaCl) and beads were resuspended in 100 μl of 2x × B&W buffer.
  8.2 100 μl purified adapter-ligated DNA was added to the beads and mixed well.
  8.3 The mixture was incubated for 15 min at RT with rotation.
  8.4 The beads were washed four times with 200 μl of 1 × B&W buffer with the addition 0.1% Tween-20, and twice with 200 μl of 10 mM Tris–HCl, pH 8.0.
  8.5 The beads were resuspended in 40 μl of 10 mM Tris–HCl, pH 8.0.
9. Library amplification were performed with KAPA HyperPlus Kits (Roche) according to the manufacturer's recommendations.

We sequenced the S1 Hi–C libraries using paired-end reads with a length of 150 bp. Read depth was 50–100 k reads per sample for shallow sequencing, ~ 37 mln reads for deeper sequenced S1 Hi–C on human K562 cells, ~ 25 mln reads for S1 digested PBMC chromatin and ~ 3.7–32.5 mln reads for K562 and digested chromatin samples.

#### S1 fragments sequencing

To prepare S1 digested chromatin libraries we followed steps 1–2.17 the S1 Hi–C protocol as described above, then proceed immediately to cross-link reversal and DNA isolation (step 5). Isolated DNA was size-selected by AMPure beads purification to remove fragments larger than 1000 bp. NGS libraries were prepared without any addition fragmentation steps. End-repairing, A-tailing, adapter ligation and library amplification were prepared using KAPA HyperPrep Kit (Roche) according to the manufacturer's recommendations.

The libraries were sequenced using paired-end reads with a length of 150 bp. Read depth was ~ 25 mln reads for S1 digested PBMC chromatin and ~ 65 mln reads for K562 and digested chromatin samples.

### Computational data analysis

#### Public data sets

The list of public data sets analyzed in this study is provided in Additional file 2: Table S1.

#### Hi–C data analysis

Hi–C data analysis and statistics computation were performed as described previously [11] with a slightly modified version of the Juicer 1.6 script and an altered computation of statistics. For all statistics computation hg19 human genome assembly was used. The script is publicly accessible on GitHub (https://github.com/genomech/juicer1.6_compact).

#### Compartmentalization

For each sample merged_nodups.txt file was generated using Juicer. This file was used to generate cool file with cooler cload pairs and cooler zoomify utils from cooler package. Thereafter, the strength of compartmentalization was calculated using cooltools. We used the same eigenvector track derived from 4DNFI18UHVRO mcool file for compartmentalization score analysis in all samples. Saddle strength profile was plotted using matplotlib.

#### CTCF sites pairs

Using gimme scan from gimmemotifs, CTCF binding sites were localized from ENCFF660GHM ChIP-seq data and MA0139.1 CTCF-motif. Thereafter, BEDPE file containing convergent pairs of the sites in the range of 50 kb–1 mb was generated.

#### Insulation score and average loop

The merged_nodups.txt from Juicer output were used to generate cool files with bins size of 5000 bp using cooler cload pairs. Then, average loops and insulation strengths were calculated using convergent CTCF pairs and CTCF peaks via tool coolpup.py (--flank 200000). Based on the resulting data, corresponding figures were constructed using plotpup.py.

#### Chromatin cut sites analysis

Sequencing data obtained in this study or downloaded from public data sets were aligned to the hg38 human genome assembly using BWA v.0.7.17–r1188. Then, bam file sorting and indexing was performed using samtools. Read-pair fragment size distributions were calculated using bamPEFragmentSize from deeptools. The distributions were plotted using matplotlib.

BigWig files containing cut site coverage were generated using bamCoverage from deeptools with the options --OffSet 1 --binSize 1.

We processed obtained bigWigs and public data describing genomic features locations in bed-format (Additional file 2: Table S1) using a python script that enables plotting of the average cleavage signal within a window of −3000 to + 3000 from the middle of the cutting sites of nuclease peaks, with (step size of 2 bp,) (refer to Fig. 4). The script is publicly accessible on GitHub (https://github.com/genomech/PlotBigwigOnBed). To ensure accuracy, we excluded genomic regions that are blacklisted by ENCODE for hg38 from the analysis (Additional file 2: Table S1). We similarly analyzed the mean S1 cleavage signal and other genomic features in the window of differentially expressed genes' transcription start sites (TSS) using K562 RNA-seq data publicly available on ENCODE (Additional file 2: Table S1). To achieve this, we defined 5% of the most abundant transcripts based on transcript per million (TPM) as highly expressed genes, and a tier of 5–20 percentile of the most abundant transcripts as intermediate expressed genes (i.e., top 20% excluding the highly expressed genes). In addition, we selected 20% of transcripts with the lowest expression levels as the non-expressed ones. Finally, we used the same script to generate plots (refer to Fig. 4).

To identify S1 sequence specificity of the S1 cut sites, cut positions were obtained from the alignment files using the following script: “*samtools view -F 16 alignment.bam | awk ‘$6* ~ *“^[0–9]* + *M” && $0 !* ~ *“MD:Z:0[ACTG]” {print $3, $4}’*” for forward reads and “*samtools view -f 16 alignment.bam | awk ‘$6* ~ *“M$” && $6* ~ *“^[0–9]*  + *M” && $0 !* ~ *“[ACTG]0[[:space:]]” && $0 !* ~ *“[ACTG]0$” {sum* = *$4* + *99; print $3, sum}’*” for reverse reads. Genomic regions flanking cut sites were extracted using pysam. Consensus logo for the extracted sequences was created using WebLogo 3.5.0.

#### Read coverage depth analysis

To analyze read coverage depth Hi–C data prepared with S1, DNase I, MNase, DpnII, and MboI enzymes were aligned with bwa mem and converted to normalized read coverage tracks (bigWig files) by deepTools 3.5.1 bamCoverage with the option --normalizeUsing RPKM [22]. To build distributions of read coverage depth genome-wide each individual chromosome in bigWig file was divided into segments of 500 nucleotides (excluding last segment), followed by calculation of the coverage sums in each segment. Analysis was performed using pyBigWig 0.3.18 [22] and NumPy 1.21.6 [23]; the histograms of distributions were plotted using matplotlib 3.5.3 [24] and seaborn 0.13.0 [25].

To compare coverage of the specific genomic elements (such as A/B-compartments or chromatin states), genomic segments for coverage sums calculation were selected according to annotation tracks. For general chromatin state segmentation we utilized publicly available GRCh37/hg19 version of Broad ChromHMM track from ENCODE/Broad [26], lifted over to hg38 human genome assembly using CrossMap 0.6.4 tool [27]. Generation of track for A and B compartments from 4DNFI18UHVRO data from 4D Nucleome Data Portal is described in Compartmentalization section of Methods. We attributed coverage sums for A or B compartment based on the value of E1 vector: genomic regions with top 15% value of E1 were assigned as A and lowest 15% were assigned as B.

Boxplots of distributions were plotted using matplotlib 3.5.3 and seaborn 0.13.0.

#### Hi–C matrices correlation analysis (reproducibility scores computation)

To assess correlation of Hi–C matrices for pairs of replicates we utilized matrices on a resolution of 100 000 base pairs with balancing weights applied (from mcool files generated as described in Compartmentalization section of Methods). For each matrix pair we calculated distribution of Spearman’s correlation coefficients by comparing row from one matrix to its corresponding row from second replicate (using cooler 0.8.5, SciPy 1.7.2 and NumPy 1.21.6 packages). Boxplots of distributions were plotted using matplotlib 3.5.3 and seaborn 0.13.0 [25].

## Results

### Boosting data yield in Hi–C protocol through DNase I substitution with S1 nuclease

Previously, we established a robust and efficient DNase I Hi–C protocol, yielding high-quality Hi–C maps [11]. Nevertheless, this method generated more dangling ends (DEs) compared to the traditional Hi–C approach. DEs represent non-chimeric DNA fragments that do not contribute valuable information for Hi–C analysis. These fragments are reduced during Hi–C library preparation through a following process: DNA ends are labeled with biotin after chromatin digestion; biotinylated nucleotide internalization following DNA end ligation; biotinylated nucleotides remaining on the unligated DNA ends are removed using exonuclease; molecules containing internal biotin (i.e., ligation products) are enriched by streptavidin pulldown. We suspect that the surplus of DEs in the DNase I Hi–C protocol is attributed to the nickase activity of DNase I. Following DNase I digestion, the 5’–3’ exonuclease activity of the Klenow Fragment elongates the nicks, incorporating biotin–dCTPs into the DNA molecule. Consequently, not only are the ligation products internally labelled with biotin, but also DNA molecules that have not participated in the ligation process.

**Fig. 1:**
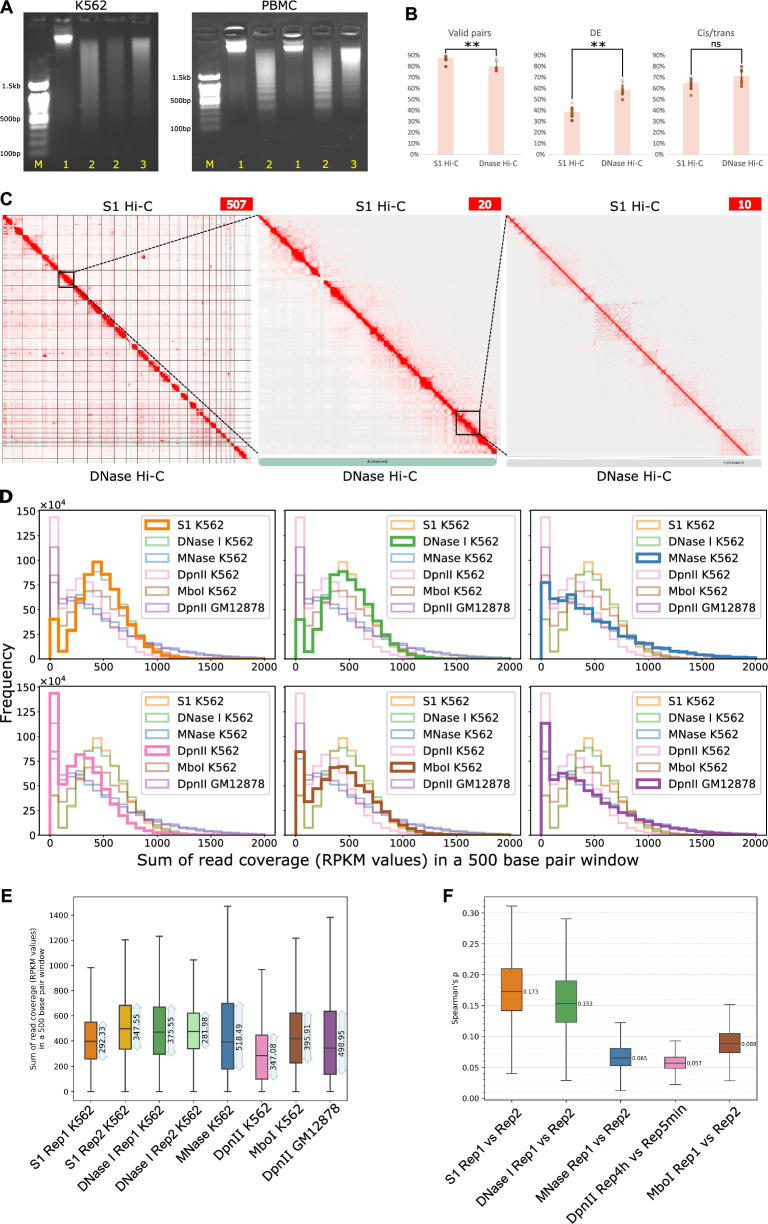
S1 Hi–C protocol allows the generation of high-quality Hi–C maps. **A** Chromatin digestion and ligation of K562 cells and peripheral blood mononuclear cells. Lanes M show a 100 bp DNA ladder. 1 — intact gDNA, 2 — S1 digestion of cross-linked chromatin, 3 — ligation of S1-digested chromatin from lane 2. **B** Quality metrics of S1 Hi–C and DNase I Hi–C data sets. Each dot represents an independent Hi–C library preparation; we analyzed 14 DNase I Hi–C libraries [11] (protocol with biotin fill-in) and 16 S1 Hi–C libraries. *P*-*values* were calculated using the Mann–Whitney test. (∗ ∗) indicates *p*-*value* < 0.01, (ns) indicates *p*-*value *> 0.05. **C** Representative heatmap of chromatin interactions in K562 cells obtained using DNase I Hi–C protocol (below the diagonal line) [11] and S1 Hi–C protocol (above the diagonal line). **D** Genome-wide read coverage depth histograms. Each histogram shows distribution of coverage depth for 500 bp genomic windows. Data were obtained by merging all replicates **E** Boxplot showing coverage distribution similar to D, but for each replicate independently. Numbers near boxplots present quantification of interquartile range. **F** Boxplot showing distribution of Spearman’s correlation coefficient, calculated between pairs of Hi–C matrices for replicates. Numbers near boxplots represent median value

Like DNase I, S1 nuclease is a sequence-agnostic nuclease capable of cleaving dsDNA, nicks, and ssDNA. We theorized that these activities of S1 nuclease could prevent the generation of nicked DNA during chromatin digestion. To verify this, we first ensured that the S1 enzyme could digest formaldehyde-fixed chromatin, producing a discernible digestion pattern (Fig. 1A). Subsequently, we modified the cell lysis and chromatin fragmentation steps (see Methods) to make the Hi–C protocol compatible with the S1 chromatin digestion. Using this modified method, we prepared S1 Hi–C libraries for 16 human peripheral blood samples and the K562 human immortalized cell line. During libraries preparation, we examined the products of digestion and ligation steps and found that they satisfy Hi–C quality standards (Fig. 1A), although we note that the pattern of chromatin fragmentation by S1 nuclease looks slightly different for different cell types (Fig. 1A).

Next, we performed shallow sequencing of these samples to assess data quality and deeper sequencing for K562 sample to produce Hi–C map (Fig. 1B, C). The quality assessment shows that compared to DNase I Hi–C, S1 Hi–C produces similarly high-quality data (Fig. 1B); however, the quantity of DEs was lower for S1 Hi–C, resulting in higher overall yield of valid Hi–C pairs. This confirms that the fraction of DE is a consequence of DNase I nickase activity, and that replacing DNase I with S1 can reduce the amount of DE fragments. In addition, S1- and DNase I-based Hi–C assays produce the smallest number of PCR duplicates compared to MNase and DpnII Hi–C data (Additional file 1: Fig. S1).

The DNase I enzyme exhibits a crucial characteristic: its ability to generate Hi–C libraries with a relatively even coverage distribution. Despite a moderate enrichment of A-compartment sequences in DNase I Hi–C libraries [28], this enrichment is less pronounced than that observed at the ends of restriction fragments in conventional Hi–C libraries. To evaluate the potential coverage bias in S1 Hi–C libraries, we computed the distribution of coverage depth across the genome in S1, DNase I, MNase, and DpnII Hi–C samples from K562 (Fig. 1D). Our findings revealed that S1, MNase, and DNase I Hi–C maintain relatively even coverage distribution. The same result can be obtained using interquartile range of coverage distribution as measure of its uniformity, as shown in Fig. 1D. In contrast, DpnII Hi–C exhibits a bimodal distribution, highlighting the disparity between sequences proximal and distal to restriction sites. Median coverage of loci attributed to A- and to B-compartment was almost similar in case of DNase I and S1 enzymes. In the MNase Hi–C data we observed a slight preference towards higher coverage of A-compartment (Additional file 1: Fig. S2A). We also performed more detailed analysis of coverage including 15 chromatin states annotated by HMM tool and did not identify any substantial bias specific to S1 enzyme (Additional file 1: Fig. 2B).

**Fig. 2:**
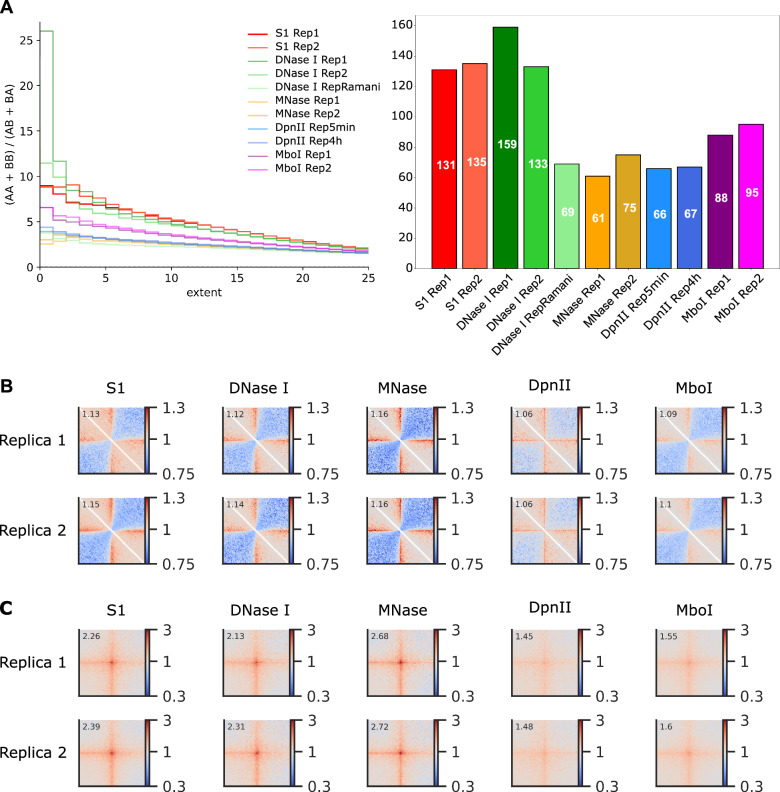
Identification of TADs, Loops and Chromatin Compartments using Hi–C data produced with different enzymes. **A** Quantification of the compartment strength using saddle plots. Left plot shows how preference of homotypic interactions (i.e., interactions within the same compartment) of the locus scales with its compartment attribution score. Right panel shows the same data in the form of a single score computed as area under the curve. **B** Aggregate TAD analysis of the Hi–C maps. The average insulation score is shown inset into each corresponding panel. **C** Aggregate loop analysis of the Hi–C maps. The average strength of loops is shown inset into each corresponding panel

We next assessed how well patterns of Hi–C data, such as TADs, loops and compartments can be detected using different enzymes. For this analysis, we again utilized Hi–C data for K562 cells, which were generated using different enzymes. As can be seen from Fig. 2, A, compared to MNase data, results obtained using S1 and DNase I provide better contrast of within vs between compartment interactions. On the other hand, TADs and loops are better resolved using MNase enzyme (Fig. 2B, C). Enzymes with specific recognition site, MboI and DpnII, generate data that is comparable with MNase in compartments analysis, and show the worst result in TADs and loop detection benchmarks (Fig. 2A–C). In addition, S1 Hi–C data show highest reproducibility scores (measured as average Spearman’s correlation between replicates) (Fig. 2F).

Finally, we assess robustness of S1 Hi–C method across cell types. For this aim, we prepared Hi–C libraries from human fibroblasts and iPS cells using the same chromatin digestion conditions as for K562 and PBMC cells. In both cases, we obtained high-quality results (Additional file 3: Table S2, Additional file 1: Fig. S1). This indicates that chromatin digestion conditions presented here are robust enough, and although optimization might be required for specific cell types, the released protocol can be used as a starting point in S1 Hi–C experiments.

Altogether, our results show that the use of S1 nuclease for chromatin fragmentation makes it possible to achieve the coverage uniformity as in DNase I and MNase Hi–C and, at the same time, to improve the quality compared to DNase I Hi–C data in terms of dangling ends fraction.

### Evaluating the cut site distribution in chromatin following S1 nuclease digestion

**Fig. 3:**
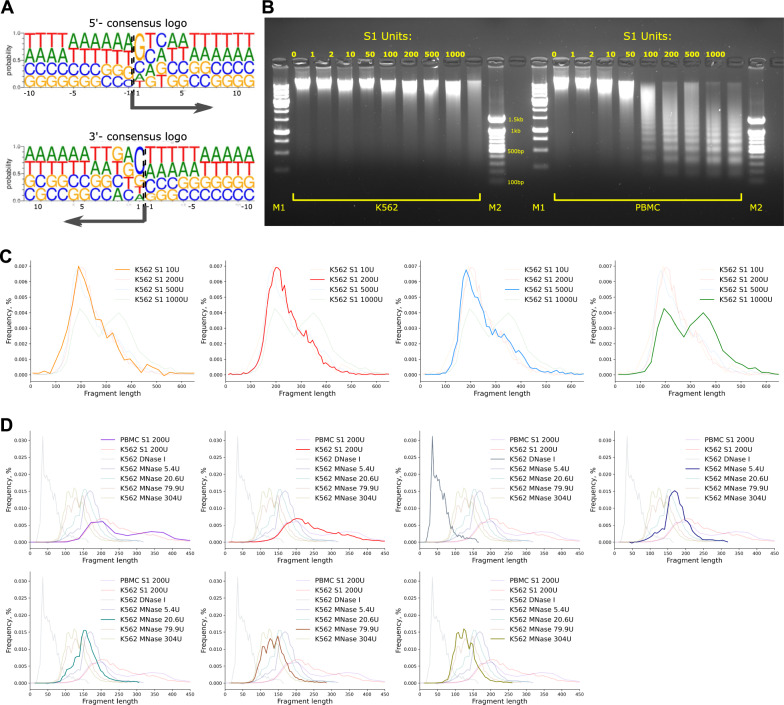
S1 nuclease chromatin digestion pattern. **A** Motif logos representing the sequence specificity of S1 nuclease cut sites. Data are shown separately for 5’-(left) and 3’-(right) ends of the digested fragments. In both cases, we show the same (reference) strand. The arrow indicates the direction of the sequencing read, and the numbers indicate the distance from the sequenced fragment end: positive numbers for internal (located within the sequenced fragment) nucleotides, negative numbers for external (located outside the sequenced fragment) nucleotides. **B** K562 and peripheral blood mononuclear cells (PBMC) chromatin digestion by different concentrations of S1 nuclease. Lanes M1 and M2 show a 1000 bp and 100 bp DNA ladders, respectively. **C** Fragment size distributions of the mapped paired-end reads for different S1 nuclease conditions in K562 cells. **D** Fragment size distributions of the mapped paired-end reads for S1 nuclease, different MNase conditions and DNase I in K562 cells

Our data suggest that S1 nuclease can be used to digest chromatin, which opens the possibility to apply S1 digestion in various chromatin profiling applications. Although the Hi–C data analysis shows that S1 digestion is fairly uniform across the genome, the complex structure of Hi–C library molecules precludes the precise identification of cut site locations. Therefore, we decided to characterize profiles of S1 nuclease digestion using fixed chromatin, which we fragmented by the enzyme and sequenced. The analysis of the obtained NGS reads revealed that the genomic fragments generated by S1 nuclease start with guanine at their 5’-end approximately two-times more frequent than expected (Fig. 3A). Thus, S1 nuclease has a slight preference to cut the primary strand immediately upstream of the guanine. Interestingly, we did not observe enrichment of cytidine in genomic position immediately before cut site, which would be expected in the case of symmetric cut (Additional file 1: Fig. S3A, B). This suggests that S1 nuclease probably cleaves DNA strands asymmetrically to form 3'-sticky ends (Additional file 1: Fig. S3A, B); the length of the overhang cannot be determined from our data. These ends could be degraded by S1 nuclease or during subsequent end repair steps of the library preparation.

To obtain a comprehensive map of DNA accessibility and understand how enzyme concentration affects digestion pattern, we treated fixed K562 cells chromatin with different concentrations of S1 nuclease: 10, 200, 500 and 1000 units. Gel-based analysis of the digestion products showed that, expectedly, higher enzyme concentration results in smaller average fragment lengths (Fig. 3B). The digestion pattern was slightly different for K562 and peripheral blood mononuclear cells (PBMC), the latter showing pronounced nucleosome-sized ladder.

To better characterize digestion profiles, we size-selected the digestion products to remove DNA fragments larger than 1000 bp and subjected the remaining DNA to NGS-library construction and paired-end sequencing. In all studied conditions, we observed clear mononucleosomal peak (~ 150–200 bp) (Fig. 3C). Interestingly, treatment of K562 chromatin with the highest S1 concentration (1000 units) results in two peaks, one corresponding to mononucleosomes (~ 150–200 bp) and another to dinuclosomes (~ 350 bp) (Fig. 3C). Besides that, there is little difference in fragment lengths distribution for studied S1 concentration (Fig. 3C). For PBMCs, we profiled single S1 concentration (200 units) and observed prominent mononuclesosmal peak accompanied by less pronounced dinucleosomal peak (Fig. 3D). The S1 fragments sizes distribution resembled the nucleosomal pattern observed for MNase, thus we reanalyzed data from [29] to compare S1 digestion pattern with the pattern produced by different MNase concentrations (Fig. 3D). MNase digests unprotected linker DNA between nucleosomes, while the DNA protected by the nucleosomes remains intact [30]. Low MNase concentrations generate fragment length distribution corresponding to mono-nucleosome-bound fragments and linker DNA. An increase in MNase concentration leads to a reduction of linker DNA due to its exonuclease activity and fragment length shift to 147 bp (Fig. 3D). Comparison of fragment length distributions suggests that S1 nuclease generates longer fragments than MNase under all conditions, arguing that it is more likely to introduce breaks between nucleosomes and has either no or reduced (compared to MNase) exonuclease activity.

**Fig. 4:**
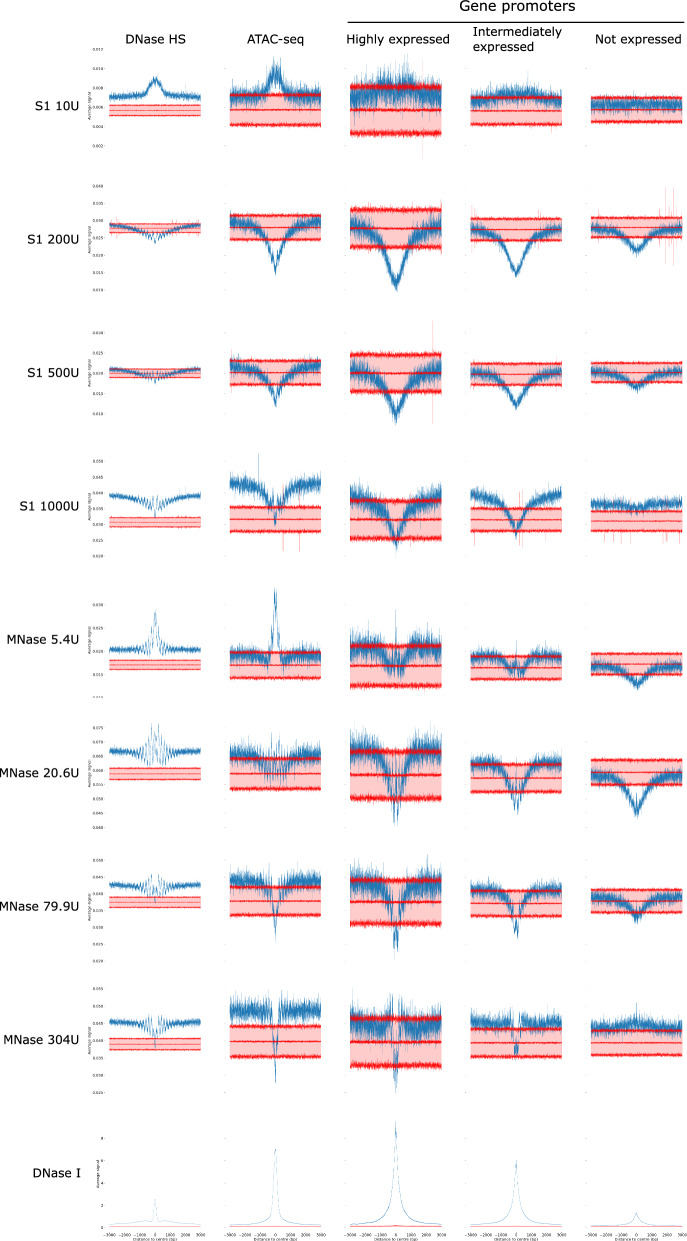
Fragment size distributions of the mapped paired-end reads and signal distributions at: ATAC-seq peaks, DNase I hypersensitive sites, and TSS for different S1 nuclease conditions, different MNase conditions and DNase I in K562 cells. Blue line shows observed signal. Red curves and shaded area between them show average + −3 standard deviations of the data obtained from 100 random shuffles of genomic feature locations (i.e., obtained by shuffling ATAC-seq peaks, DNase I hypersensitive sites or gene promoters)

Next, we aggregated the S1 nuclease, DNase I, and MNase DNA break location frequencies across annotated open chromatin features: ATAC-seq peaks and DNase I hypersensitive sites (HS) in K562 cells. As DNase I HS and ATAC-seq peaks both align with cis-regulatory elements, such as promoters and enhancers of actively transcribed genes, the aggregation of cut sites for these enzymes displays a high degree of concordance (Fig. 4). The location of MNase cut sites is dependent on enzyme concentration: at low concentrations, the signal heightened across open chromatin regions, implying that these were the first sites accessible to the enzyme. Conversely, at higher MNase concentrations, open chromatin regions were depleted due to elevated digestion of accessible chromatin. The pattern observed for S1 enzyme across DNase I or ATAC-seq peaks also shows similar trend. For low S1 concentration, we detect enrichment around open chromatin regions, whereas for higher concentration we observe reduced signal in the middle of the peak, followed by gradual increase with nucleosomal pattern (waves). This signal resembles the pattern observed for moderate or high MNase concentrations; however, nucleosomal pattern was less pronounced than for high MNase concentration, suggesting that S1 nuclease may not have the strong exonuclease activity required to digest linker DNA.

Nucleosome positioning and chromatin accessibility shows non-random pattern across gene promoters, with the level of accessibility correlating with the gene expression. We analyzed the chromatin cut frequency across transcription start sites (TSS) stratifying genes by the expression level (Fig. 4). Both S1 nuclease and MNase show decreased fragment ends frequency near TSS of actively expressed genes, presumably because these regions are accessible to enzyme and over-digested, producing very small fragments (or even digested into individual nucleotides), that cannot be captured by sequencing. The reduced signal region was broader in S1 nuclease data than in MNase data, and does not show a clear nucleosome pattern.

Drawing from these findings, we speculate that S1 nuclease exhibits greater activity on DNA bound to nucleosomes than MNase, while its exonuclease activity to digest linker DNA is either lower or non-existent. This results in a more uniform distribution of S1 nuclease cut sites in comparison with MNase (which is additionally supported by analysis of Hi–C reads coverage distribution presented in Fig. 1E). In relation to DNase, S1 nuclease presents a decreased representation of fragment ends within open chromatin regions, a pattern that may be attributable to the presence of exonuclease activity, or possibly due to high endonuclease activity that reduces these loci into fragments too minuscule for detection. Finally, S1 nuclease shows slight preference towards cleavage of guanine 5-phosphate bonds, leaving a 3’-overhang on the complementary strand. Despite these preferences, the overall pattern of S1 cuts is relatively uniform (compared to DNase I or MNase digestion) and thus allows studying both open and closed chromatin.

## Discussion

Here, we confirm that S1 nuclease [31] can be used to cut fixed chromatin and show that this property of enzyme can be utilized for Hi–C libraries preparation. Sequence-agnostic digestion with S1 nuclease is important for Hi–C applications, including genome assembly or structural variants detection, where the resolution of breakpoints is limited by a frequency of genomic location of digestion sites. We developed a robust and efficient protocol for S1 Hi–C analysis of PBMC, which can be used to capture structural variants in cells and tissues in humans. Future applications can extend S1 Hi–C protocols for genome assembly and haplotyping.

Our analysis of S1 digestion products provides detailed information for developing of future genomic assays based on S1 enzyme. We note that this assay is based on short-read sequencing and includes size-selection procedure. Therefore, our analysis is biased towards relatively small (< 1 kb) genomic fragments. Within this fragment length, S1 nuclease shows relatively uniform digestion profile generating mainly nucleosome-sized fragments.

### Supplementary Information

**Additional file 1: ** Supplementary Figures S1-S3.

**Additional file 2: Table S1. **The list of public data sets analyzed in this study is provided in Additional file.

**Additional file 3: Table S2. **Data quality metrics.

## Data Availability

Sequencing data generated in this study are accessible via the NCBI BioProject PRJNA985640.
