# Supplementary material for: Expanding the list of sequence-agnostic enzymes for chromatin conformation capture assays with S1 nuclease
Source: Epigenetics Chromatin. 2023 Dec 11;16:48. doi: 10.1186/s13072-023-00524-4 (PMC10712037; doi:10.1186/s13072-023-00524-4)

Supplementary Figure 1. Selected statistics of Hi-C data produced with different enzymes on K562 cells.

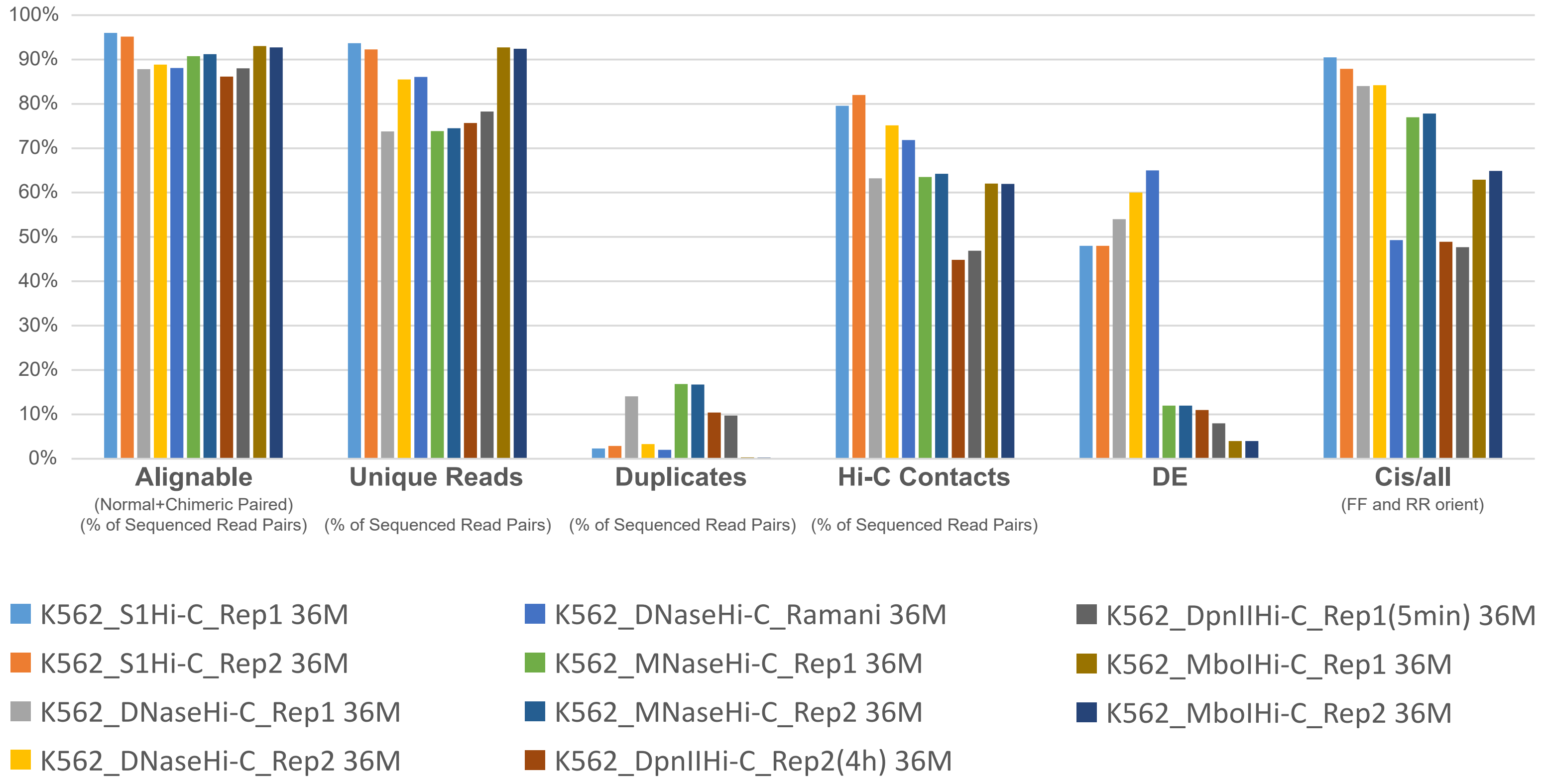

Supplementary Figure 2. Read coverage depth histograms showing distribution in annotated regions. (A) A/B compartments. (B) Chromatin state segmentation by Broad ChromHMM track.

A

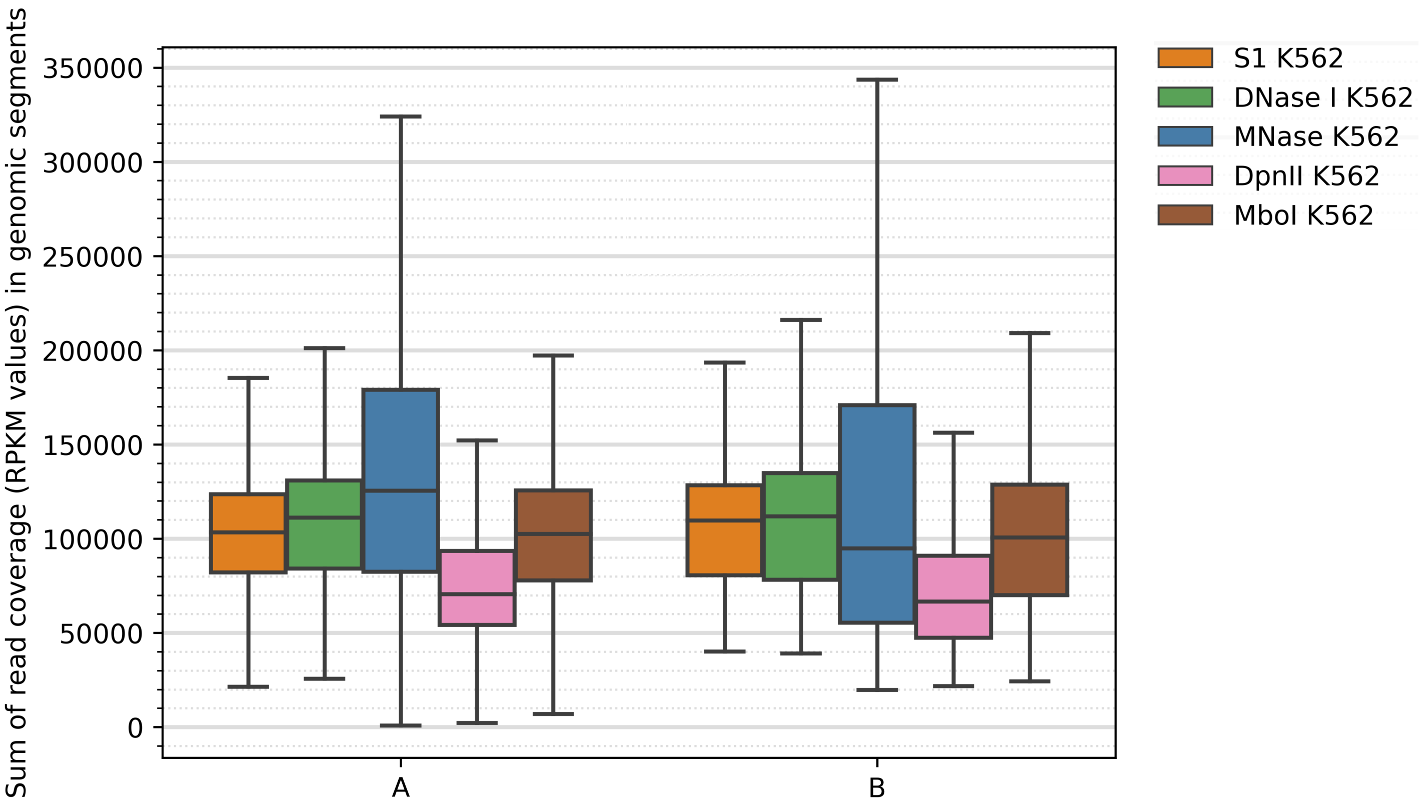

B

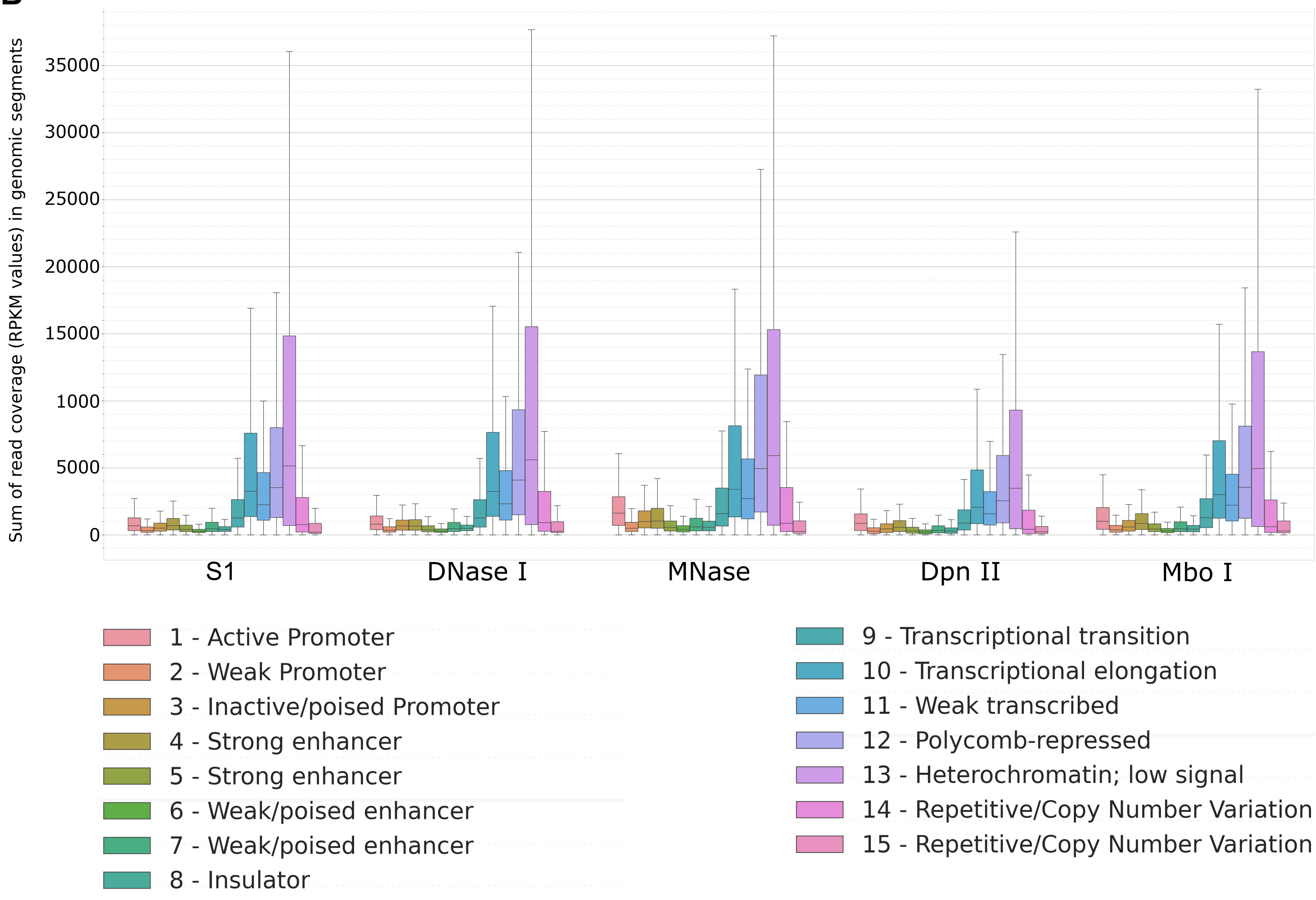

Supplementary Figure 3. Schematic representation of S1 nuclease digestion. S1 nuclease introduces a double strand break at the sites (red arrowhead) resulting in blunt ends (A) or 3'-sticky ends (B). In the case of S1 nuclease makes blunt ends, we would expect C in -1 position of 50% forward reads and G in -1 position of 50% reverse reads (the indicates distance to the sequenced fragment end, i.e. +1 is a first nucleotide within sequenced fragment and -1 is a first letter in the flanking genomic sequence). This does not correspond to the obtained data. In the case of S1 nuclease makes 3'-sticky ends, they will be resected by S1 or during the next steps of library preparation. As a result, in -1 position of forward and reverse reads, we would find random nucleotides. This is concordant with the obtained data.

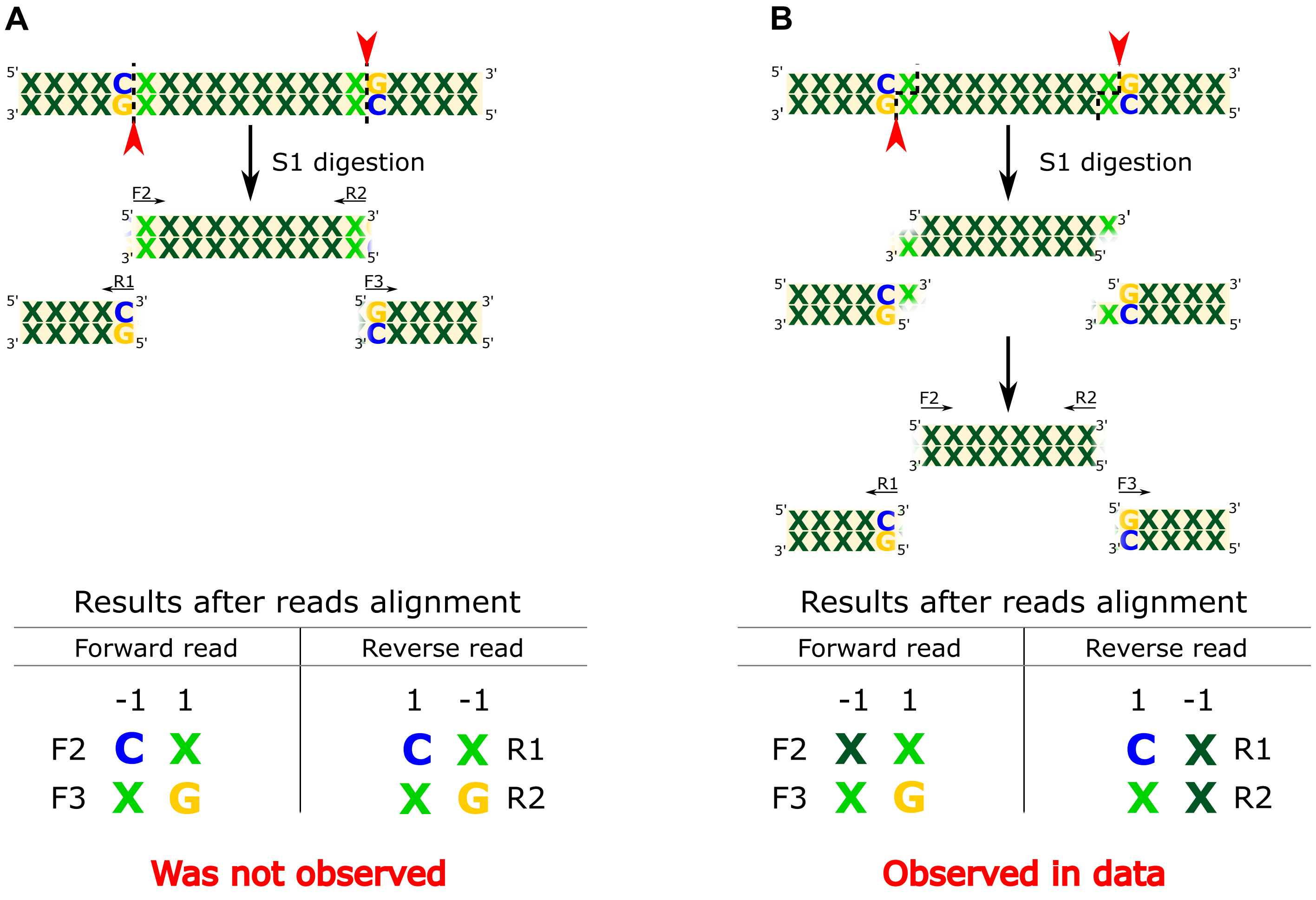

Supplement: Supplementary file 1 — Additional file 1: Supplementary Figures S1-S3. [file 13072_2023_524_MOESM1_ESM.pdf]
